# Characterization of RNase MRP RNA and novel snoRNAs from Giardia intestinalis and Trichomonas vaginalis

**DOI:** 10.1186/1471-2164-12-550

**Published:** 2011-11-06

**Authors:** Xiaowei S Chen, David Penny, Lesley J Collins

**Affiliations:** 1 Department of Biochemistry, University of Otago, Dunedin, New Zealand; 2 Institute of Molecular Biosciences, Massey University, Palmerston North 4442, New Zealand; 3 Institute of Fundamental Sciences, Massey University, Palmerston North 4442, New Zealand

## Abstract

**Background:**

Eukaryotic cells possess a complex network of RNA machineries which function in RNA-processing and cellular regulation which includes transcription, translation, silencing, editing and epigenetic control. Studies of model organisms have shown that many ncRNAs of the RNA-infrastructure are highly conserved, but little is known from non-model protists. In this study we have conducted a genome-scale survey of medium-length ncRNAs from the protozoan parasites *Giardia intestinalis *and *Trichomonas vaginalis*.

**Results:**

We have identified the previously 'missing' *Giardia *RNase MRP RNA, which is a key ribozyme involved in pre-rRNA processing. We have also uncovered 18 new H/ACA box snoRNAs, expanding our knowledge of the H/ACA family of snoRNAs.

**Conclusions:**

Results indicate that *Giardia intestinalis *and *Trichomonas vaginalis*, like their distant multicellular relatives, contain a rich infrastructure of RNA-based processing. From here we can investigate the evolution of RNA processing networks in eukaryotes.

## Background

The current view of cellular RNA organization indicates an RNA infrastructure [1,2], which describes the spatial and temporal network of the many different RNAs. The interconnection of the RNA-processing pathways is crucial for cellular processes because different RNA processing mechanisms are tightly linked. For example, transcription and splicing happen in close proximity [3,4], and splicing is tightly connected with downstream mRNA processes including localization, translational regulation, and nonsense-mediated decay [5-7]. With the advancement of large-scale RNA analysis and high-throughput sequencing, conserved features of the eukaryotic RNA infrastructure have come to light from studies in animals and plants. In contrast to the genome-wide transcriptional information known in other eukaryotic models, only limited information on RNA biology is available from *G. intestinalis *and *T. vaginalis*. We can compare the ncRNAs from these two protists with other model eukaryotes (e.g. animals, plants, yeasts), to understand more about the general nature of the RNA infrastructure within eukaryotes.

**Figure 1:**
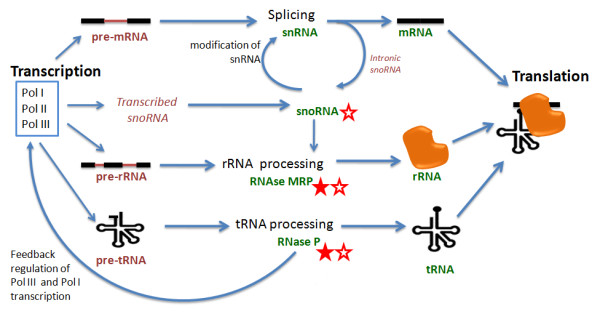
**Central Processing RNA-infrastructure of eukaryotes**. This representation of the central processing RNA-infrastructure of eukaryotes indicates where processing ncRNAs from *G. intestinalis *(filled star) and *T. vaginalis *(unfilled star) are unclear either in identification or annotation. We note that intronic snoRNAs are highly unlikely in both of these species because their introns are very short and there are so few of them. Figure adapted with permission from Collins 2011 (*RNA infrastructure and networks*).

Previous studies in *Giardia *have identified key eukaryotic ncRNAs such as the RNase P [8], snoRNAs [9,10], spliceosomal snRNAs [11], miRNAs [12-14] and antisense transcripts [15]. Studies on *Trichomonas *ncRNAs show that the currently known ncRNAs also exhibit typical features of eukaryotes [8,16-18] including RNase P [8], RNase MRP [8], snRNAs [17] and some snoRNAs [9,14]. However, there are still gaps in our knowledge of RNA processing in these species, especially in the characterization of the RNase MRP RNA in *Giardia*, and the different types of snoRNAs in *Trichomonas *(Figure 1). Annotations of the RNase P RNA are also not clear in these genomes. In this study, we characterize medium length ncRNAs including RNase MRP, RNase P and snoRNAs from two protozoan parasites: *Giardia intestinalis *(Diplomonad) and *Trichomonas vaginalis *(Parabasalid), to clarify uncertainties about conserved features of the RNA infrastructure.

We have previously sequenced ncRNAs sized from 10 to 200 nucleotides (nt) from *G. intestinalis *and *T. vaginalis*, and identified from this data small ncRNAs such as microRNAs in the two protists [14]. Using this same sequencing data we are also able to analyze longer ncRNAs due to the wide size range. In this study we identify medium-length ncRNAs (50-250 nt) from small RNA based sequencing data, characterize the RNase MRP of *G. intestinalis*, and clarify annotations of RNase P and MRP from both *G. intestinalis *and *T. vaginalis*. We also identify new H/ACA box snoRNAs from these species. Our study clearly demonstrates that high-throughput sequencing cannot only screen for small regulatory RNAs, but can also be used for the characterization of longer ncRNAs from diverse organisms. Results from our work support that *G. intestinalis *and *T. vaginalis *possess a rich network of RNA processing components expected in the consensus eukaryotic RNA infrastructure.

## Results

### Construction of RNA contigs using consensus mapping

The total RNAs from *G. intestinalis *and *T. vaginalis *were purified, size fractionated and sequenced according to Methods. Constructing contigs of short to medium RNAs requires a different approach from standard assembly protocols. Most ncRNAs do not have long poly-A tails; therefore small-RNA sequencing is a way of recovering medium-length ncRNAs when used in combination with consensus mapping. We successfully generated RNA contigs from *G. intestinalis *and *T. vaginalis *and compared the updated mapping software Bowtie [19] to the original software Maq [20]. Overall, the contigs constructed using Bowtie were longer compared to the ones constructed using Maq, although the overall number of contigs was fewer (Table 1).

**Table 1:** Summary of RNA consensus contig results

	*G. intestinalis* 1	*G. intestinalis* 2	*T. vaginalis* 1	*T. vaginalis* *2*
**Mapping software**	Maq	Bowtie	Maq	Bowtie
**Consensus calling**	Maq-assemble	mpileup*	Maq-assemble	mpileup*
**Number of Contigs**	7051	6521	18310	3787
**Mean length (nt)**	45	92	42	100
**Median length (nt)**	36	68	36	79
**Max length (nt)**	439	6017	352	522

*mpileup is part of the SAMTOOLS package

With the length cutoff of the new contig datasets set above 50nt we discarded mature miRNAs and siRNAs. *De novo *assembly tools such as Velvet [21] and Abyss [22] produced very few contigs and therefore were not used in this study. Hence, we recommend that for medium length ncRNA assembly that a reference genome is used for the initial assembly until tools are developed to permit the *de novo *assembly of small contigs. Our study was carried out on *G. intestinalis *WB strain (Genome Assemblage A), and the genome assemblages of the other two strains (Isolate GS/Assemblage B, and Isolate P15/Assemblage E) were used for comparison in some of the subsequent analysis.

Our RNA contigs and trimmed sequences were compared to the available *G. intestinalis *and *T. vaginalis *genome annotation to check that rRNAs and tRNAs were represented as expected. From the *G. intestinalis *sequences all 80 annotated rRNAs and tRNAs from the WB isolate were covered by contigs generated with Bowtie mapping. However, many of these contigs contain Illumina short reads mapped to multiple sites in the genome and therefore were assigned equally to each of the possibilities. Contigs were trimmed in length from the 5' end, to a minimum length of 20 nt, 36 nt and 50 nt to use as sequence tags and mapped against the annotated ncRNAs. Our original trimmed sequence datasets were also mapped against the annotated ncRNAs. All *G. intestinalis *and *T. vaginalis *rRNAs were found by sequence and contigs. In *G. intestinalis *all tRNAs and rRNAs were found from Assemblage A with 3 tRNAs not found by contig but found by sequence in Assemblage B, (P15) (Trp, Met, Phe) and four not found by sequence or contig (Cys, Tyr, His, Asp). A comparison against Assemblage E (Isolate P15) had 4 tRNAs (Trp, Met, Cys, Asp) not found by sequence or contig. Given that our sequences came from the same strain as Assemblage A, not finding some sequences in the other strains is not surprising. *T. vaginalis *had many annotations for its rRNAs and tRNAs and the majority were found by both contig and sequence (results are summarized in Additional file 1, Tables s1-s3).

Results from comparing our contigs and sequences with known tRNAs and rRNAs indicate that our method is assembling ncRNAs effectively, and that such contigs can be used as sequence tags if trimmed to 20 or 36 nucleotides.

### Identification of the MRP RNA from *G. intestinalis*

RNase MRP is a ribonucleoprotein complex, consisting of one ncRNA and (in humans) ~10 proteins. It catalyzes the nucleotide cleavage reaction at the A3 site (at the internal transcribed spacer region between small-subunit and 5.8S rRNAs) on the pre-rRNA transcript. MRP RNA is evolutionarily related to the eukaryotic RNase P RNA which processes the 3'- end of pre-tRNA transcripts [23]. The RNase P and MRP complexes share a number of common protein subunits [24,25], and the secondary structures of RNase P and MRP RNAs share a common backbone. The RNase P RNA has been identified in all eukaryotes and prokaryotes studied to date and shown to be one of the few RNAs that have retained catalytic features [26,27]. The RNase MRP is thought to have evolved before the ancestor of modern eukaryotes [28], but previously missing evidence of the MRP RNA from *G. intestinalis *[8,29] imposed a question on the origin of MRP and rRNA processing. However the conserved A3 site on rRNA-gene sequence has been identified in *G. intestinalis*, strongly suggesting the presence of *G. intestinalis *MRP RNA [30].

**Figure 2:**
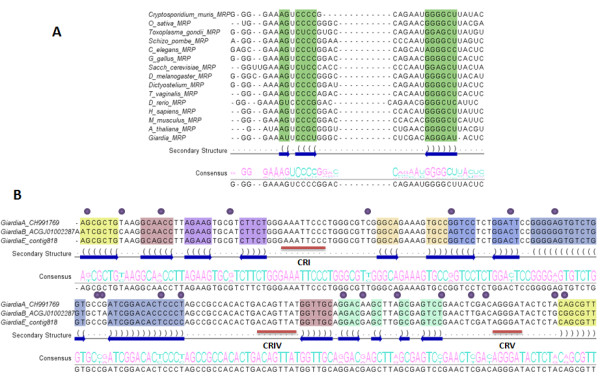
***G. intestinalis *RNase MRP RNA sequence alignment analysis**. **A**. The consensus MRP RNA backbone structure. **B**. Multiple sequence alignment of CR-1 and CR-5 regions from eukaryotic species showing a high degree of similarity in these regions. **C**. Multiple sequence/structure alignment of *G. intestinalis *MRP RNAs from Assemblage A (CH991769:781009-781197), B (ACGJ01002287:25810-25999) and E (contig188:88192-88380). Each stem structure is coded in a different color and positions of nucleotide substitution are indicated by dots.

Our contigs contained a sequence that with Infernal 1.0 [31] mapped to the conserved CR-1 consensus region of the P4 pseudoknot structure of the MRP core structure (Figure 2A). Both the pseudoknot and the actual sequences of CR-1 and CR-5 regions have previously been shown to be evolutionarily conserved throughout most eukaryotes [8]. Sequence alignment of the CR-1 and downstream predicted CR-5 regions of *G. intestinalis *MRP and a representative of other eukaryotic MRPs showed a high degree of sequence similarity (Figure 2B).

Upon further analysis, contigs upstream and downstream to the conserved CR-1 region were found that did not have enough overlap to permit a longer contig to be predicted computationally. This region is highly conserved between the three isolates of *G. intestinalis *(Assemblages A, B and E) and as expected, our sequence had 100% identity to isolate A (Figure 2C). Comparing the alignment with the predicted secondary structure (Figure 2C) indicates that half of the nucleotide differences occur in single stranded regions and all but one change at the beginning of helix P1 are either compensated with a change at the corresponding position or changed to permit G-U wobble pairing. Thus, it is unlikely that any of these changes will have a major impact on the overall RNase MRP RNP secondary structure. All predicted RNase MRP RNA and RNase P RNAs from *G. intestinalis *and *T. vaginalis *are given in Table 2.

**Table 2:** Genomic location of *G. intestinalis *and *T. vaginalis *RNase MRP and RNase P RNA genes

	Assemblage	Strain	Contig	Co-ordinates
**RNase MRP RNA**	Giardia A Giardia B Giardia E	Isolate WB Isolate GS Isolate P15	CH991769 ACGJ01002297 contig818	781009-781197 (-) 25811-25999 (+) 88192-88390 (-)
	
	*T. vaginalis*	Strain G3	DS114691 DS113339	8345-8566 (-) 56271-56492 (+)

**RNase P RNA**	Giardia A Giardia B Giardia E	Isolate WB Isolate GS Isolate P15	CH991762 ACGJ01002916 contig173	145450-145695 (+) 916-1152 (-) 30157-30402 (+)
	
	*T. vaginalis*	Strain G3	DS113188 DS114246	290253-290503 (+) 14234-14484 (-)

All genomes downloaded from GiardiaDB (version 2.3) and TrichDB (version 1.2) [45]

**Figure 3:**
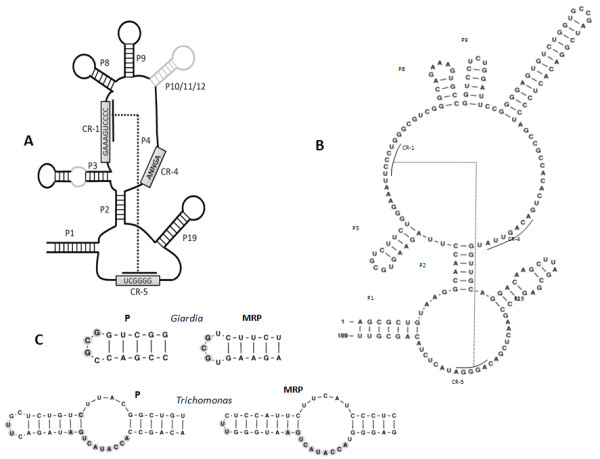
**RNase MRP RNA secondary structure analysis**. **A**. The secondary structure of *G. intestinalis *RNase MRP RNA. **B**. The secondary structure of *T. vaginalis *RNase MRP RNA. **C**. Analysis of the P3 helix. The P3 helix is shorter and lacks the internal loop but is comparable with what is found in the *G. intestinalis *RNase P RNA. *Trichomonas *also has a conserved region shared between the P3 helices of RNase P and RNase MRP RNA.

The entire predicted secondary structure of *G. intestinalis *MRP including these conserved regions is shown in Figure 3A. A closer look at the structure shows that the CR-4 region also has the previously identified conserved pattern "ANAGNNA" [32] where the three most conserved "A"s are present. Not unexpectedly given the reduced nature of the *G. intestinalis *genome, the length and structure of the *G. intestinalis *MRP RNA makes it one of the shortest among studied eukaryotes. In *T. vaginalis *the MRP RNA is longer and has a more extended P3 helix (Figure 3B). Structurally, the *G. intestinalis *MRP RNA is closer to that of the microsporidium species such as *Nosema locustae *and *Encephalitozoon cuniculi*, which are known to have the shortest MRPs [28,32].

Another important characteristic of the MRP RNA is the P3 helix, which is a structure common between RNase MRP and P. The P3 helix associates with the protein POP1 [33], and for MRP it is essential for the cleavage of pre-rRNAs at the A3 site on the pre-ribosomal RNA transcript [33]. Previous computational studies have already identified both the RNase P and RNase MRP RNAs from *T. vaginalis *[8]. The P3 helix of *G. intestinalis *RNase P and RNase MRP are both short and lack the intra-helical loop, however they do share a 3 nt sequence at the terminal loop, consistent with previous studies showing the same relation of P and MRP in a number of eukaryotes [8,28]. Comparison of the P3 helices of *T. vaginalis *RNase P and MRP also show a consensus sequence located at the intra-helical loop and the terminal loop regions (Figure 3C). The structure of helix P19 between *G. intestinalis *MRP and P is also conserved, although no sequence similarity is observed.

Although we are concentrating on the RNase P and RNase MRP ncRNA molecules from *G. intestinalis *and *T. vaginalis*, it is worth a quick mention of the important proteins associated with their ribonucleoprotein macromolecules. RNase P and RNase MRP macromolecules share many of their proteins [24,25,34], which have been characterized to small extent in *G. intestinalis *[35]. Some proteins such as the scaffolding protein POP1[33] can be hard to identify in protists, due to the large amount of evolutionary distance between them and species from which these proteins are known, but other proteins such as POP4 are much more conserved across eukaryotes [35]. For a more detailed analysis of the changes in the secondary structure of the RNA components we will require further domain and protein structural analysis to understand how the RNA and Protein components have evolved together. However, even without this detailed protein-RNA analysis, we can see that our results strongly support the conservation of the structure-function relationship between RNase MRP and P within *G. intestinalis *and *T. vaginalis*.

Our study also sought to clarify the annotation of the RNase MRP RNA especially in the Assemblage B from version 2.3 of the genome. In this annotation within contig ACGJ00100236, the MRP RNA is overlapping with a snoRNA which is in turn overlapping an area annotated as RNase P. The RNase MRP RNA from *G. intestinalis *we have identified does not match this region, but instead this region corresponds to its close relative the RNase P RNA. Corrected contigs and co-ordinates for the Assemblage B genes are given in Table 2.

Although we have characterized the *G. intestinalis *MRP RNA computationally, further molecular biology experimentation will be required before it can be functionally verified. Until then, we classify our sequences in *G. intestinalis *as computationally predicted.

### New H/ACA box snoRNAs

Small nucleolar (sno)RNAs are a group of ncRNAs of variable length (from 60 up to 1000 nt in yeast), which are involved in processing of several types of transcripts [36]. Most of the known snoRNAs belong to two classes, which are determined by evolutionarily conserved sequence elements: the C/D box and H/ACA box [36]. SnoRNAs exist in large numbers in eukaryotes. In humans, there have been more than 400 snoRNAs identified [29], and in general have been shown to have a diverse range of locations and expression patterns [37]. In our previous studies, we have characterized novel C/D box snoRNAs in both *G. intestinalis *and *T. vaginalis *[9,14]. C/D box snoRNAs direct 2'-O methylation, and are relatively easy to identify based on conserved sequence elements and complementary binding to the target RNAs. H/ACA box snoRNAs direct pseudouridylation, and often exhibit more variable features due to their shorter length of conserved elements and discontinuous complementary target binding regions.

**Figure 4:**
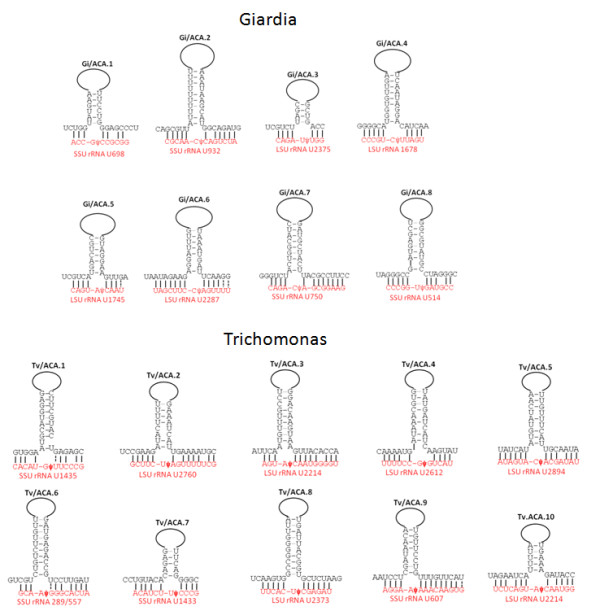
**snoRNA-rRNA binding structures of the new H/ACA box-snoRNAs**. The new snoRNAs from *G. intestinalis *are named Gi/ACA.x and those from *T. vaginalis *are named Tv/ACA.x. The proposed pseudouridylation sites are marked by "ψ". The detailed structures of the stems are now shown here (simplified by the loops).

As a first step in identification of new H/ACA box snoRNAs, the rRNAs of *G. intestinalis *and *T. vaginalis *were aligned with human rRNAs to locate conserved pseudouridylation sites, which were then incorporated into the search for the complementary target-binding regions in candidate H/ACA box snoRNAs. After generating the negative controls, the RNA contigs were searched using the established parameters from control runs (see Methods). Table 3 shows the scoring rules and results from subsequent analysis. In total, 8 *G. intestinalis *and 10 *T. vaginalis *new snoRNAs were identified using this method. The RNA sequences and corresponding genomic positions are in Additional file 2 and Additional file 3. One set of the new *G. intestinalis *snoRNAs (Gi/ACA.1 and Gi/ACA.2), and two sets of the new *T. vaginalis *snoRNAs (Tv/ACA.1 and Tv/ACA.2; Tv/ACA.6 and Tv/ACA.7) are overlapping in the same genomic region on the plus and minus strand respectively. The structures of the target binding regions of new snoRNAs are shown in Figure 4. Candidates had either one or two stems, but all contain only one target-binding site. Candidates with two stems can have the target-binding site on either stem. Further analysis showed that their second stems do not have targets on rRNAs despite having the conserved stem-loop structure upstream of the ACA box.

**Table 3:** Scoring rules and scores of snoRNA candidates

Model	Percentage†	Cutoff score	Number of candidates*	Average of candidates' total score	Max score	Min score
***G. intestinalis***						
**2 stem**	95.23%	26	7	29.33	32.32	27.61
**1 stem**	95.94%	20.5	1	23.79		

***T. vaginalis***						
**2 stem**	95.20%	25.5	7	28.36	25.91	30.08
**1 stem**	95.98%	20.5	3	23.67	21.11	26.61

†The percentage of control sequences with total scores below the cutoff score

*Number of predicted candidates with total scores above the Cutoff score set from control analysis

The new *G. intestinalis *snoRNAs all have different target pseudouridylation sites on rRNAs, whereas two of the *T. vaginalis *new snoRNAs (Tv/ACA.3 and Tv/ACA.10) share the same target (LSU rRNA U2214). In addition, Gi/ACA.5, Tv/ACA.3 and Tv/ACA.10 target a conserved site on the large subunit rRNA, the same as Gi/ACA.6 and Tv/ACA.2. The target regions are highly conserved between *G. intestinalis *and *T. vaginalis*, but the sequences of the snoRNAs targeting these sites do not show substantial sequence similarity. A BLAST [38] search of the newly identified *G. intestinalis *H/ACA box snoRNAs was performed against the genomes of the other two isolates of *Giardia *strains (Assemblage B and E) to look for homologous sequences. Three *G. intestinalis *new snoRNAs have homologous sequences in either Assemblage B or E, and 2 have homologous sequences in both Assemblage B and E. Overall the sequences are highly conserved and the nucleotides complementary to the rRNA target sequences show minimal changes across the three *Giardia *strains.

## Discussion

Recent studies on ncRNAs throughout eukaryotes have expanded our understanding of the RNA-processing infrastructure [1] with the discovery that key components of the RNA-processing machinery occur throughout eukaryotes. It is now clear that the general ncRNA infrastructure has been conserved in excavates, which is an extended but less studied group of eukaryotes.

Characterization of the *G. intestinalis *RNase MRP RNA is an important achievement in searching for conserved key ncRNAs of the central RNA-processing pathway. Sequence and structural analysis of the *G. intestinalis *MRP RNA has shown all the conserved characteristics of eukaryotic MRP, as referred to in Figure 3. The conserved structural relationship between *G. intestinalis *P and MRP RNAs indicates that the protein-RNA relation in *G. intestinalis *P and MRP does not differ significantly from other eukaryotes. Identification of the MRP RNA from *G. intestinalis *has filled the gap left from previous studies of MRPs. In looking at the structure of the *G. intestinalis *MRP we can see how consensus models based on the eukaryotic P3 region could not have detected either the sequence or the structure. The typical P3 region of *G. intestinalis *resembles more the bacterial model of RNase P RNA than the eukaryotic P3 model for both RNase P and RNase MRP RNAs. However, the rest of the structure fits the eukaryotic RNase MRP RNA model. This demonstrates that with protists in particular, the standard eukaryotic models for ncRNAs may not necessarily apply.

The novel H/ACA box snoRNAs identified from *G. intestinalis *and *T. vaginalis *all have only one predicted target-binding site regardless the number of stem-loops. Having one target-binding site is also seen in the H/ACA box snoRNAs found in Trypanosomes [39]. Identification of the new snoRNAs is usually reliant on predicted conserved target sites, which however appear only partially conserved across distant organisms. ncRNAs in protists may exhibit characteristics not typically seen in more commonly studied model species, and thus these methods may not reveal all the ncRNAs within non-model genomes. For instance the number of pseudouridylation sites in Trypanosomes is estimated to be 70-80 [39], but to date only around 50% of snoRNAs involved in the modification of these sites have been found [40]. Therefore we can expect that the total number of H/ACA box snoRNAs will actually be much larger in both *G. intestinalis *and *T. vaginalis*. Understanding more about general structural and sequence motifs, will aid us in further searches for H/ACA box snoRNAs.

**Figure 5:**
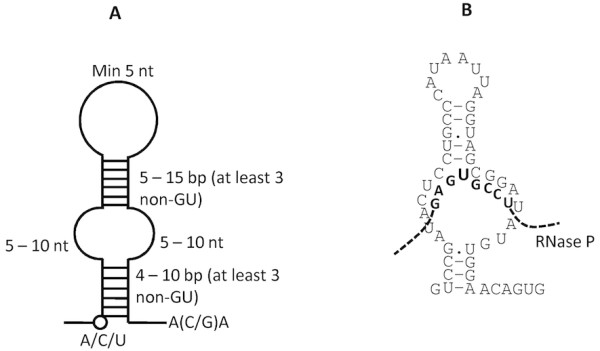
**H/ACA-like RNAs in *G. intestinalis***. **A**. RNAmotif model for searching H/ACA-like RNA motifs: This model is based on the common structure of the first stem-loop of *G. intestinalis *H/ACA-box snoRNAs identified in this study. **B**. An H/ACA-like RNA with a possible RNase P target. Structural analysis shows strong complementary binding between an H/ACA-like RNA and the *G. intestinalis *RNase P RNA.

Many snoRNAs do not have identified targets and are therefore termed orphan snoRNAs [37]. Although there have not been extensive studies, there is no reason to believe that similar orphan snoRNAs will not exist in protists. A conserved single stem-loop structure constructed by RNAMotif [41] was tested to look for possible H/ACA-like motifs in *G. intestinalis *contigs (Figure 5A). Results show 29 RNA contigs adopted the model, and two of them overlap with the SnoGPS-identified candidates. However, without the constraint of pre-specified target sequences, the predicted structures are not identical. Interestingly, one of the H/ACA-like RNAs shows a possible target on the *G. intestinalis *RNase P RNA (Figure-5B). However, despite strong G-C rich complementary binding, the potential editing site does not have an un-bound nucleotide immediately 3' to the uridine, as typically observed in all H/ACA box snoRNAs found so far. Furthermore, there are only 3 base pairs on the 5' side of the base-pairing region instead of 4 to 10 base pairs typically observed in known snoRNAs. In addition, there has been no evidence of pseudouridine modification in RNase P. Therefore, any conclusion that this ncRNA is acting as an orphan snoRNA on RNaseP cannot be made at this stage. Apart from the above, the rest of these 29 H/ACA-like RNAs do not appear to have rRNA targets.

We demonstrate in our study that high-throughput sequencing of ncRNAs larger than ~21-25nt (typical for miRNA and siRNA studies) is possible, and that the assembly of short read sequences can lead to the characterization of medium-length ncRNAs. We have found this technology to be a boost to the study of ncRNAs from non-model eukaryotes and especially those distantly related to well characterized species (e.g. human, mosquito, nematode, yeasts and some plants). A limitation of this study is that the sequencing length of 36nt (standard in ncRNA sequencing) is short and that longer sequences would enable the identification of ncRNAs ~70-150nt in single reads, thus not requiring assembly.

## Conclusions

In conclusion, we constructed new RNA contigs with updated genomes and identified the RNase MRP of *G. intestinalis*, which answers positively the previously open question as whether MRP exists in all extant eukaryotes with the A3 site. In addition, a number of new H/ACA box snoRNAs have been identified in *G. intestinalis *and *T. vaginalis*, with a reduced structure compared to model species, but still possessing the characteristic target-binding pattern and sequence motifs. It is becoming evident that not only are the components of RNA-processing network highly conserved within eukaryotes, but also the pattern of transcription across the genome appears to be shared among distant lineages. Overall, our results imply that it is increasingly likely that the main classes of RNA processing and regulation were present in the last common ancestor of eukaryotes [42]. We demonstrate that high-throughput sequencing can be used for the characterization of ncRNAs longer than 21-22nt small regulatory RNAs for which, in ncRNA studies, this technology is typically applied.

Our general strategy has been to search for the major classes of RNA in all major groups of eukaryotes and investigate the evolution of their mechanisms [14,43,44]. Increasingly it appears that the major groups are universal in eukaryotes, even though there is continued evolution of individual subgroups of regulatory RNA. Discovering how RNA systems work in protists, which are distantly remote in an evolutionary sense from other eukaryotes, may hold the key in uncovering how RNA mechanisms evolved from our early ancestors.

## Methods

### Cultures and RNA sequencing

Note that the sample preparation and sequencing was prepared as for previous publications from this data [14]. We have summarised this procedure here.

*G. intestinalis *trophozoites (WB strain) were grown in TY1-S-33 media, and *T. vaginalis *was maintained in T. vaginalis broth (Fort Richard) at 37°C. Cells were harvested by centrifugation (2,500 rpm, 10 min, 4°C). Growth media was removed and cells were washed once in PBS buffer. Total RNA was extracted using Trizol (Invitrogen) according to the protocol provided by the manufacturer, and further purified by phenol: chloroform extraction. The purified RNA was dissolved in double distilled water. For sequencing, 10 μg of DNase treated, 5'- de-capped total RNAs were separated on a 15% denaturing acrylamide 8 M urea gel and RNAs ranging from 10 to 200 nt were cut out from the gel and prepared according to Illumina's small RNA preparation protocol. This effectively includes an RT-PCR step that converts RNA to DNA for further sequencing. 8 and 12 pmol (in each lane) of *G. intestinalis *and *T. vaginalis *cDNA were sent for sequencing on an Illumina Genome Analyzer for 36 cycles. Pipeline analysis was performed with the Illumina Pipeline version 1.4.

### RNA Consensus Contig construction

During our previous study [14] full length (36nt) trimmed (22nt) and unique read datasets were mapped to the genomes [45] of *G. intestinalis *(version 1.2) and *T. vaginalis *(version 1.1) and short consensus 'RNA contigs' were generated using Maq version 0.7.1 [20]. We initially used these RNA contigs, then constructed new RNA contigs generated when updated genomes became available for downloading from GiardiaDB and TrichDB [45] (*G. intestinalis *version 2.3 and *T. vaginalis *version 1.2). For these new contigs we used Bowtie [19] for mapping, and SAMTools [46] for conversion and consensus sequence generation. Contigs less than 50nt were discarded for this study. A covariance model of MRP RNA was built from the seed alignment of 89 MRP RNA sequences from the Rfam database [29], and then used to search for MRP candidates in *G. intestinalis *RNA contigs using Infernal 1.0 [31]. Secondary structures were drawn using VARNA [47] and the potential RNase MRP was compared to similar regions in the other *Giardia *strains using BLAST[38].

### snoRNA search and characterisation

The search for new H/ACA box snoRNAs used SnoGPS 2.0 [48] with default parameters and predicted pseudouridylation sites. The negative control sequences for SnoGPS were generated using uShuffle [49], and additional analysis was carried out using RNAMotif [41] to look for H/ACA-like RNA structures. Potential rRNA modification sites needed to be clarified before searching for new H/ACA-box snoRNAs from *G. intestinalis *and *T. vaginalis*. rRNAs of *G. intestinalis *and *T. vaginalis *were first aligned with human rRNAs, then conserved sites in rRNAs with known pseudouridylation in human were selected as possible target sites. These sites were then used for snoRNA prediction using SnoGPS [48]. Initially, SnoGPS control runs were performed with randomized sequences in order to determine the distribution of total scores for later analysis runs. To construct these random sequences, a selection of *G. intestinalis *and *T. vaginalis *contigs were shuffled with the same nucleotide frequency 100 times and the resulting sequences were used as control input for SnoGPS program. First, the standard two stem-loop model was used, and this search permitted G-U base pairs in snoRNA-target binding. However, previous studies have shown that snoRNAs in *G. intestinalis *could also adopt an archaeal one-stem structure [9], therefore, a one-stem search was also tested. The total score cutoff for searching the original contigs was set to above 95% of the randomized control sequences.

All new sequences are in preparation to be included into Rfam [29].

## Authors' contributions

XSC carried out the culturing and RNA isolation, ran the snoRNA analysis and drafted the manuscript. LJC carried out the MRP analysis, coordinated the project and helped to draft the manuscript. DP conceived of the study, and participated in its design and helped to draft the manuscript. All authors read and approved the final manuscript.

## Supplementary Material

Additional file 1 **Supplementary Tables**. Click here for file

Additional file 2 **G. intestinalis H/ACA snoRNAs**. Click here for file

Additional file 3 **T. vaginalis vaginalis H/ACA snoRNAs**. Click here for file
