# Supplementary material for: Characterization of RNase MRP RNA and novel snoRNAs from Giardia intestinalis and Trichomonas vaginalis
Source: BMC Genomics. 2011 Nov 6;12:550. doi: 10.1186/1471-2164-12-550 (PMC3228867; doi:10.1186/1471-2164-12-550)
Supplement: Additional file 1 — Supplementary Tables. [file 1471-2164-12-550-S1.PDF]

Supplementary Tables

Key: ***** low numbers of sequences hit this gene. **a** longer contigs had a greater success than shorter ones (i.e a contig from contigs36 could distinguish the tRNA and map when the equivalent contig from contig20 did not.

Table s1 Mapping of trimmed contigs and sequences against rRNAs of Giardia Assemblage A

rRNA	# of Annotated genes	# found contigs20	# found contigs36	# found contigs50	# found sequences
**5 S rRNA**	1	1	0	0	1
**5.8S rRNA**	1	1	0	0	1
**18 S rRNA**	4	4	2	2	4
**28 S rRNA**	4	3	1	0	4

Table s2 Mapping of trimmed contigs and sequences against tRNAs of Giardia Assemblage A

tRNA	# of Annotated genes	# found contigs20	# found contigs36	# found contigs50	# found sequences
**Ser**	4	4	4	2	4
**Val**	4	2	1	1	4
**Asn**	2	2	2	2	2
**Arg**	6	3	4a	3a	6
**Trp**	1	0	0	0	1*
**Met**	3	1	1	0	3
**Ala**	4	2	1	1	4
**Gly**	4	2	1	1	4
**Glu**	3	2	1	1	3
**Gln**	4	2	2	2	4
**Pro**	3	1	1a	1a	3
**Ile**	4	3	2	1	4
**Thr**	3	3	2	1	3
**Leu**	6	3	4a	4a	6
**Cys**	1	0	1a	1a	1
**Tyr**	1	1	1	1	1
**Phe**	1	0	1a	1a	1
**Lys**	3	2	1	1	3
**His**	1	0	1a	1a	1
**Asp**	1	2	2	2	1

Table s3 Mapping of trimmed contigs and sequences against tRNAs of Trichomonas

tRNA	# of Annotated genes	# found contigs20	# found contigs36	# found contigs50	# found sequences
Ala AGC	19	13	0	0	17
CGC	2	2	0	0	2
TGC	7	6	0	0	5
Arg ACG	16	14	3	2	16
CCG	2	1	1	1	2
CCT	4	4	2	1	4
TCG	1	1	1	1	1
TCT	8	5	1	0	8
Asn GTT	17	12	9	12	17
Asp GTC	26	17	1	1	25
Cys GCA	12	9	0	0	11
Gln CTG	9	6	5	6	9
TTG	9	6	5	3	9
Glu CTC	14	9	5	2	14
TTC	24	16	6	5	24
Gly CCC	3	1	0	0	2
GCC	18	10	11	9	18
TCC	8	5	0	0	7
His GTG	10	6	7	7	10
Ile GAT	20	18	3	2	20
TAT	10	8	2	1	9
Leu AAG	19	11	0	0	19
CAA	5	2	0	0	3
CAG	2	2	1	1	2
TAA	9	5	2	0	7
TAG	2	2	0	0	2
Lys CTT	20	14	13	13	20
TTT	19	17	9	5	19
Met CAT	22	14	2	1	22
Phe AAA	2	2	1	1	2
GAA	15	12	6	6	15
Pro AGG	3	3	0	0	3
CGG	2	2	2	1	2
TGG	11	9	0	0	11

**Ser ACT**	2	1	1	1	1
**AGA**	12	8	1	1	1
**CGA**	3	3	1	1	1
**GCT**	5	3	3	4	5
**GGA**	1	1	0	1	1
**TGA**	7	5	1	2	6
**Thr AGT**	5	4	2	2	5
**CGT**	2	2	0	0	2
**TGT**	18	11	2	1	18
**Trp CCA**	5	5	0	0	5
**Tyr GTA**	13	11	6	5	13
**Val CAC**	1	1	1	1	1
**GAC**	21	10	7	7	21
**TAC**	3	3	3	2	3
